# Supplementary material for: Lymphatic dysfunction in lupus contributes to cutaneous photosensitivity and lymph node B cell responses
Source: J Clin Invest. 2025 Apr 22;135(12):e168412. doi: 10.1172/JCI168412 (PMC12165800; doi:10.1172/JCI168412)
Supplement: Supplemental data [file jci-135-168412-s043.pdf]

Supplemental Figures 1-6 for Howlader, Ambler et al.

Lymphatic dysfunction in lupus contributes to cutaneous photosensitivity and lymph node B cell responses

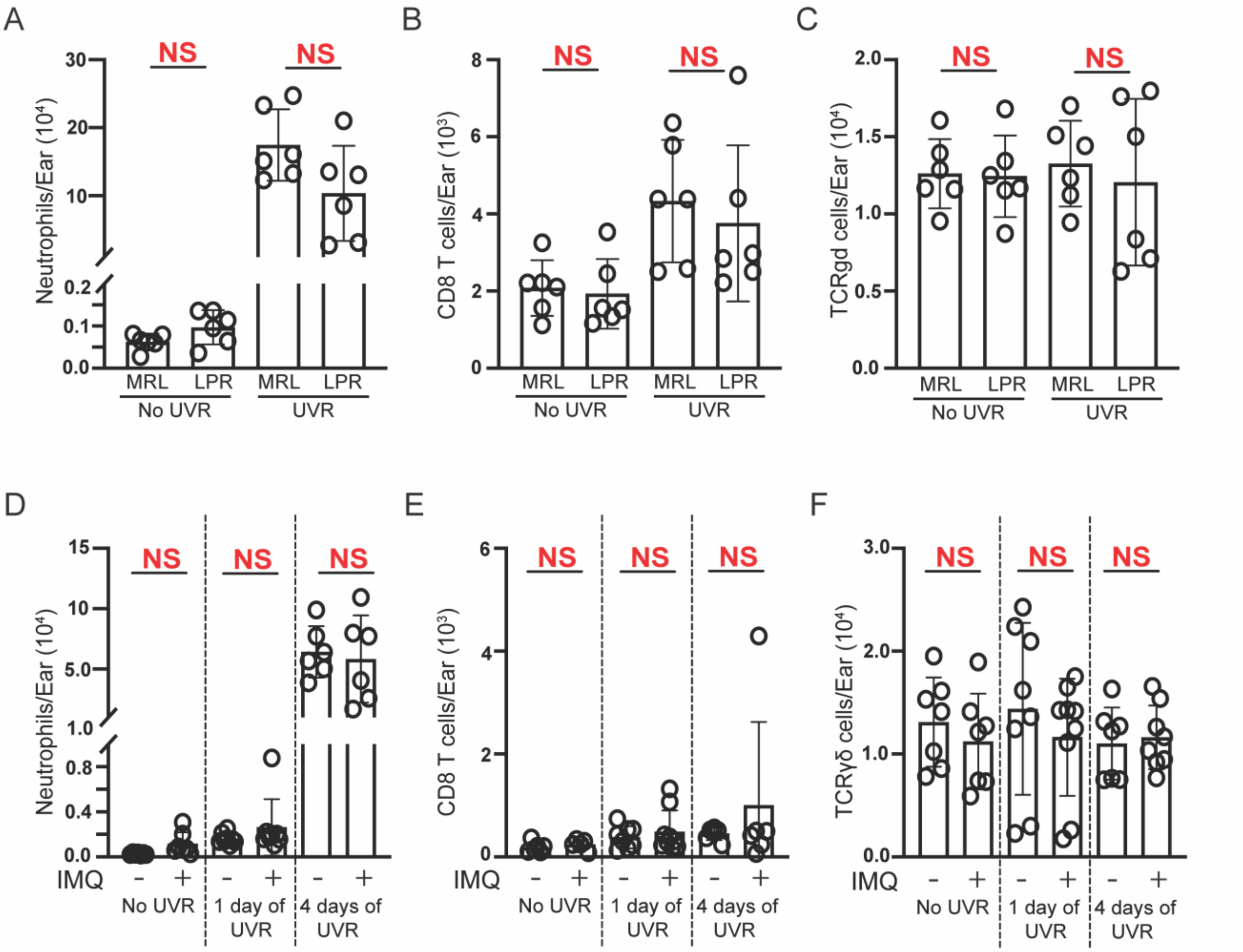

Supplemental Figure 1. LPR and B6-IMQ mice show no changes in neutrophil, CD8 T cell, and TCRγδ T cell numbers in left ear after UVR exposure

(A-C) LPR mice with no UVR or treated with 4 days UVR had left ears collected 24 hours after final UVR dose. (A) Neutrophil, (B) TCRαβ CD8 T cell, (C) TCRγδ T cell numbers. (D-F) B6-IMQ mice were treated with zero, 1 day, or 4 days of UVR prior to left ear harvest. (D) Neutrophil, (E) TCRαβ CD8 T cell, and (F) TCRγδ cell numbers. Each symbol represents one mouse; n= 5 to 9 per condition; data are from 2 (A-C), 8 (E, F), and 11 (D) independent experiments. The Shapiro-Wilk test was used to test for normality. Unpaired t test was used for normal data and Mann-Whitney U test was used otherwise. ***P<0.001, **P<0.01, *P<0.05, NS=not significant. Error bars represent SD.

A

Apoptosis pathways that are different between IMQ painted and unpainted ears	FDR	log2 FC
REACTOME_APOPTOTIC_CLEAVAGE_OF_CELL_ADHESION_PROTEINS	4.16E-05	0.44
REACTOME_DEFECTIVE_INTRINSIC_PATHWAY_FOR_APOPTOSIS	4.99E-05	0.30
REACTOME_APOPTOTIC_EXECUTION_PHASE	1.92E-04	0.15
REACTOME_APOPTOTIC_CLEAVAGE_OF_CELLULAR_PROTEINS	2.63E-03	0.12
REACTOME_APOPTOSIS	2.64E-03	0.10
REACTOME_CYTOCHROME_C_MEDIATED_APOPTOTIC_RESPONSE	2.16E-02	0.08
REACTOME_INTRINSIC_PATHWAY_FOR_APOPTOSIS	2.87E-02	0.07

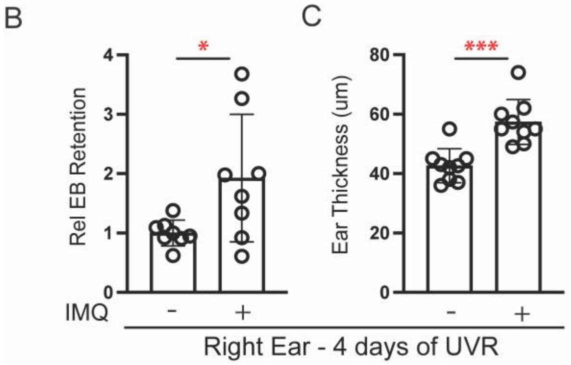

Supplemental Figure 2. Direct IMQ exposure of the painted right ear in B6-IMQ mice upregulates damage pathways, and UVR exposure results in impaired lymphatic flow and increased swelling of right ear

Tissue apoptotic pathways are upregulated in IMQ-painted (right) ear compared to unpainted (left) ear in the B6-IMQ model. Bulk RNA seq data of IMQ painted and unpainted ear skin were previously published (1). This dataset was queried for significantly different expression levels of apoptotic pathways in the Reactome Pathway Database, and the results are shown. (B-C) B6-IMQ mice were treated with 4 days of UVR and painted right ear was assessed for (B) Evans blue dye retention and (C) ear thickness. Each symbol represents one mouse; n= 8 to 9 per condition; data are from 4 (B) and 6 (C) independent experiments. The Shapiro-Wilk test was used to test for normality. Unpaired t test was used for normal data and Mann-Whitney U test was used otherwise. ***P<0.001, **P<0.01, *P<0.05, NS=not significant. Error bars represent SD.

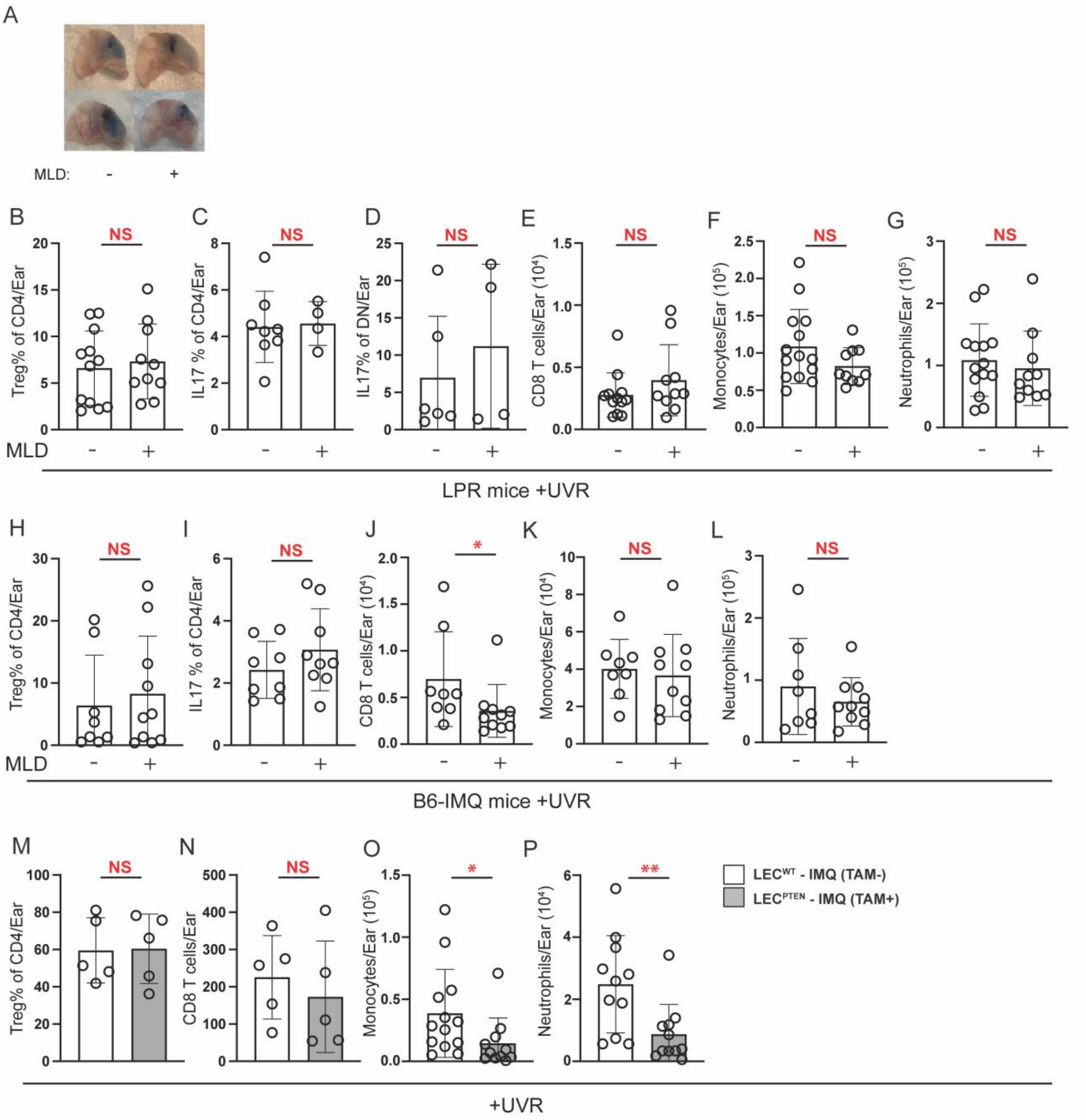

Supplemental Figure 3. Additional assessment of immune cell changes in skin with improved lymphatic flow

(A) Ears illustrating visible changes in Evans blue dye retention with MLD. Left ears of LPR mice that received 4 days of UVR +/- MLD were injected with Evans blue dye at 1 day after final UVR dose and ear harvested 1 day later. Left ears of (B-G) LPR and (H-

L) B6-IMQ mice that received UVR exposure with or without MLD for 4 days were examined by flow cytometry. (B, H) Percentage of TCR$\alpha\beta$ CD4 T cells that were Foxp3+CD25+ Tregs and (C, I) IL-17+ Th17 cells. (D) Percentage of DN cells expressing IL17. (E, J) CD8 T cell, (F, K) monocyte, and (G, L) neutrophil numbers. (M-P) Left ears of LEC^{PTEN}-IMQ and LEC^{WT}-IMQ mice that received 4 days of UVR exposure were examined similarly to above for (M) Treg percentage of CD4 T cells and (N) CD8 T cell, (O) monocyte, and (P) neutrophil numbers. Each symbol represents one mouse; n= 4 to 14 per condition; data are from 2 (C, D, M, N), 3 (H-L), 4 (B, E-G), and 5 (O, P) independent experiments. The Shapiro-Wilk test was used to test for normality. Unpaired t test was used for normal data and Mann-Whitney U test was used otherwise. ***P<0.001, **P<0.01, *P<0.05, NS=not significant. Error bars represent SD.

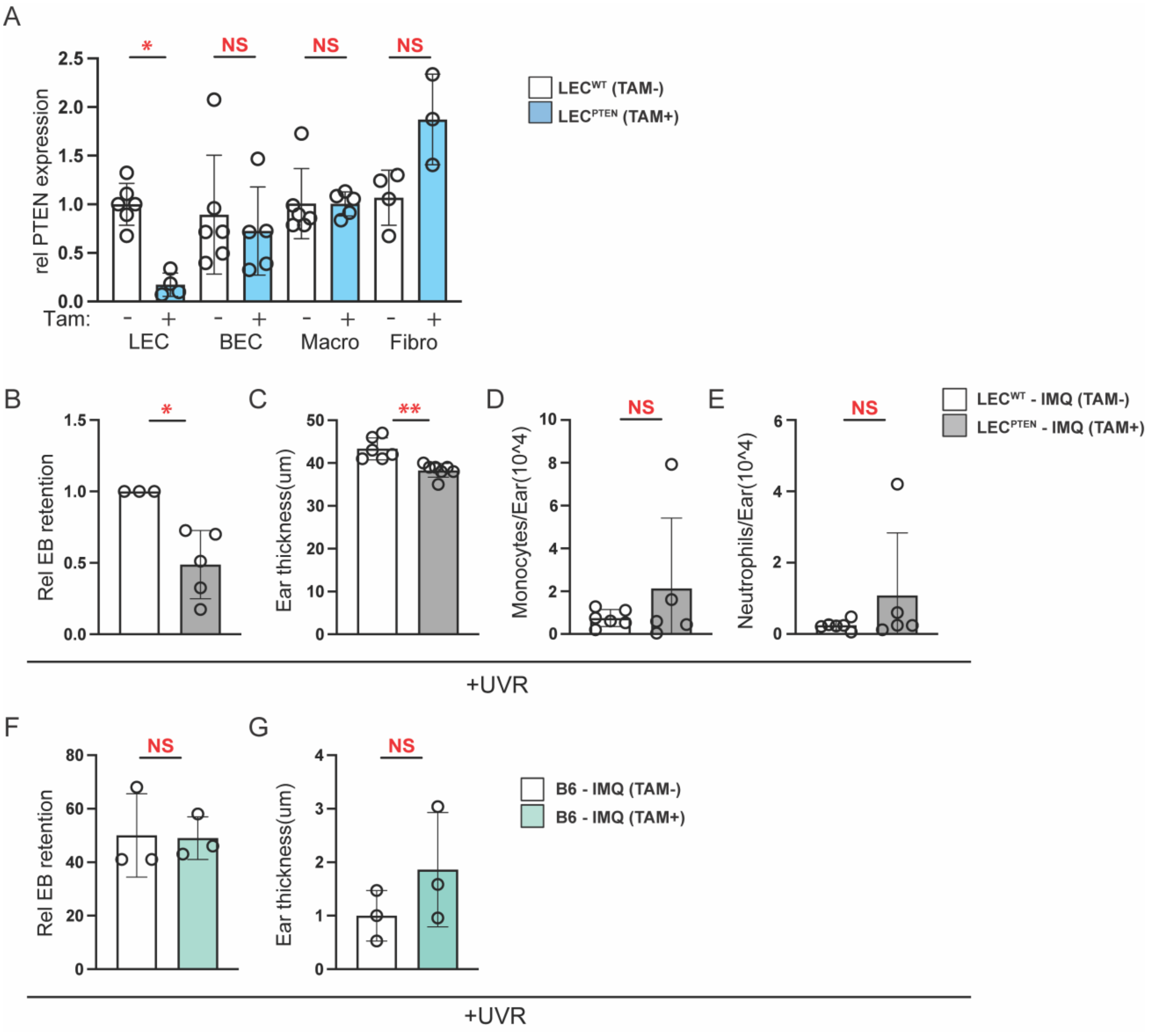

Supplemental Figure 4. Tamoxifen-treated Flt4Cre^{ERT2}PTEN$^{fl/fl}$ mice had selective knockout of PTEN in LECs and right ear assessment in LEC^{PTEN}-IMQ mice and associated controls

(A) PTEN expression as determined by qPCR of lymphatic endothelial cells (LECs), blood endothelial cells (BECs), macrophages, and fibroblasts sorted from ear skin of Flt4Cre^{ERT2}PTEN$^{fl/fl}$ mice +/- tamoxifen. (B-G) LEC^{PTEN}-IMQ and LEC^{WT}-IMQ mice (B-E) and B6-IMQ mice +/- tamoxifen (F, G) were treated with 4 days of UVR and IMQ-

painted right ears were assessed for Evans Blue retention (B, F), ear thickness (C, G), monocytes (D) and neutrophils (E). Each symbol represents one mouse; n= 3 to 7 per condition; data are from 2 (D-G), 3 (A, B), and 4 (C) independent experiments. The Shapiro-Wilk test was used to test for normality. Unpaired t test was used for normal data and Mann-Whitney U test was used otherwise. ***$P<0.001$, **$P<0.01$, *$P<0.05$, NS=not significant. Error bars represent SD.

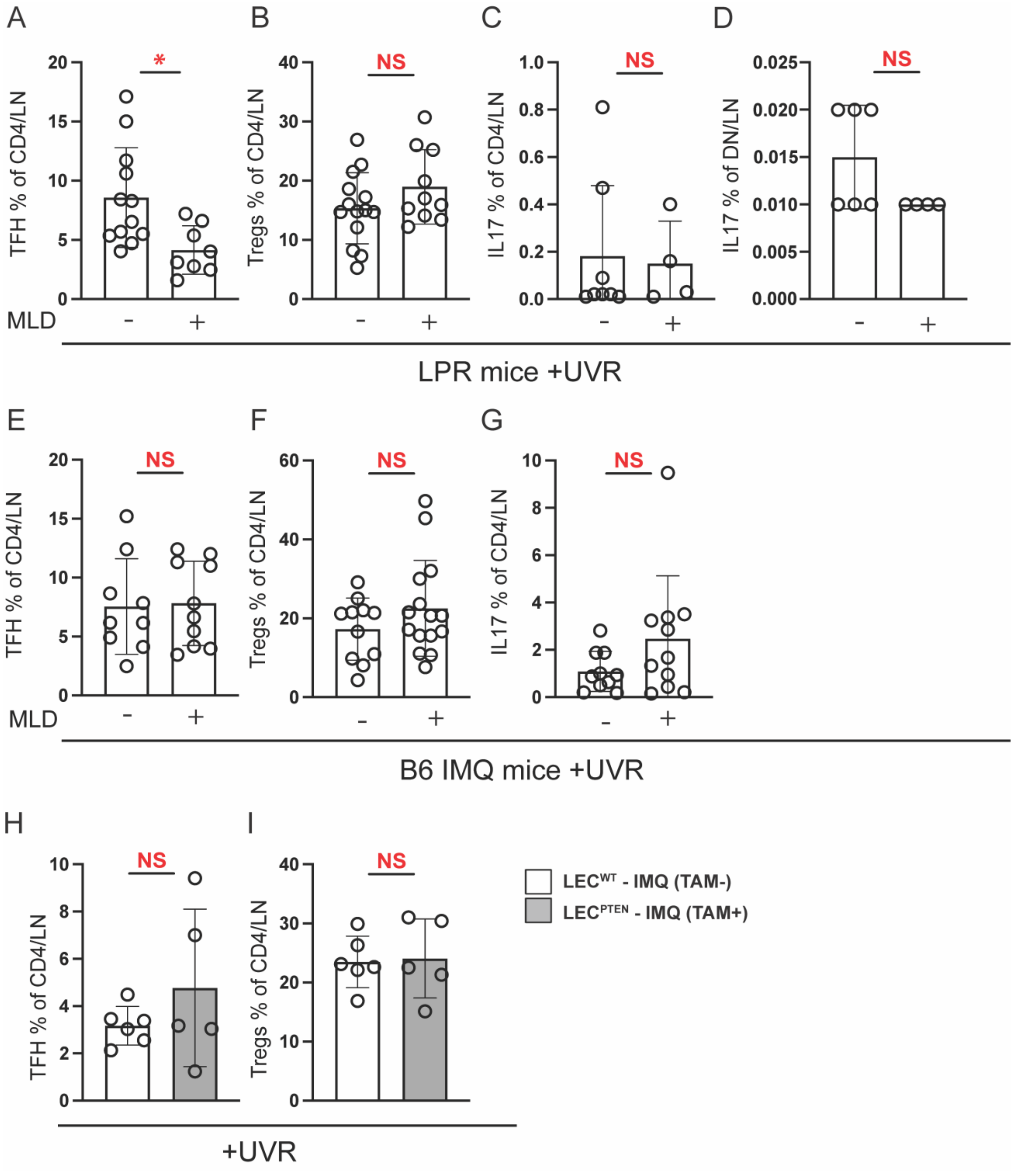

Supplemental Figure 5. Additional assessment of immune cell changes in draining lymph nodes with improved lymphatic flow

(A-G) Left auricular nodes of LPR (A-D) and B6-IMQ (E-G) mice that received UVR +/- MLD for 4 days were examined by flow cytometry. (A,E) T follicular helper, (B,F) Treg, and (C, G) Th17 cells, as a percentage of TCRαβ+CD4+ T cells. (D) Percentage of TCRαβ+CD3+CD4-CD8- DN T cells that express IL-17. (H-I) Left auricular lymph nodes of LEC^{PTEN}-IMQ and LEC^{WT}-IMQ mice were examined for (H) TFH and (I) Treg cells as a percentage of CD4 T cells. Each symbol represents one mouse; n= 4 to 15 per condition; data are from 2 (C, D, H, I), 3 (A, E, G), and 4 (B, F) independent experiments. The Shapiro-Wilk test was used to test for normality. Unpaired t test was used for normal data and Mann-Whitney U test was used otherwise. ***P<0.001, **P<0.01, *P<0.05, NS=not significant. Error bars represent SD.

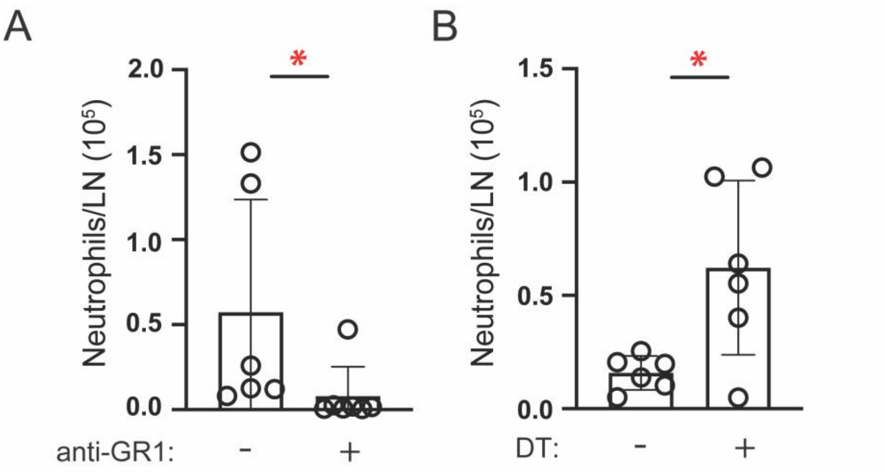

Supplemental Figure 6. Neutrophils are depleted with anti-Gr-1 and not depleted with DT treatment of CCR2-DTR mice.

(A) B6-IMQ mice were treated with anti-Gr-1 or isotype control at days -1, 0, and +2 of UVR and MLD treatments and left auricular lymph node was examined for CD11b+Ly6C+Ly6G+ neutrophil numbers. (B) CCR2-DTR mice painted with IMQ to induce the IMQ model were treated with DT at days 0, and +2 of UVR and MLD treatments, and left auricular lymph node was examined for neutrophil numbers. Each symbol represents one mouse; n= 6-7 per condition; data are from 3 (A) and 5 (B) independent experiments. The Shapiro-Wilk test was used to test for normality. Unpaired t test was used for normal data and Mann-Whitney U test was used otherwise. ***P<0.001, **P<0.01, *P<0.05, NS=not significant. Error bars represent SD.

Name	Source	Catalogue #
CD3e APC-Cy7	Biolegend	100330
B220 PE-Cy7	Biolegend	103222
gp38 PE-Cy7	Biolegend	127412
gp38 APC-Cy7	Biolegend	127418
Ly6C PE-Cy7	Biolegend	128018
Ly6C BV785	Biolegend	128041
CD45.2 APC-Cy7	Biolegend	109824
CD45 PerCP-Cy5.5	Biolegend	103132
CD45 AF700	Biolegend	103128
CD45 BV785	Biolegend	103149
CD11b BV605	Biolegend	101257
CD11b PE-Cy7	Biolegend	101216
CD11c APC-Cy7	Biolegend	117324
CD11c PE	Biolegend	117308
Ly6G APC-Cy7	Biolegend	405208
Ly6g AF700	Biolegend	127622
GL7 Pacific blue	Biolegend	144614
Peanut Agglutinin (PNA), Biotinylated	Vector Laboratories	B-1075-5
CD138 APC	Biolegend	142506
IgG1 FITC	BD Biosciences	553443
IgG2a/2b FITC	BD Biosciences	553399
IgG3 FITC	BD Biosciences	553403
Ig kappa FITC	Southern Biotechnologies	1170-02S
Ki-67 AF647	BD Biosciences	558615
CD31 Pacific Blue	Biolegend	102422
CD31 PerCP-Cy5.5	Biolegend	102420
CD4 APC-Cy7	Biolegend	100414
CD8a PerCP-Cy5.5	BD Biosciences	551162
TCR beta AF700	Invitrogen	56-5961-82
TCR g/d PE-Cy7	Biolegend	118124
CD25 PE	Biolegend	102008
CD25 BUV395	BD Biosciences	564022
FOXP3 AF488	Biolegend	126406
CXCR5 BV785	Biolegend	145523
CD279 (PD-1) BV711	Biolegend	135231
IFN gamma (IFNg) PE	Ebioscience	12-7311-82
IL-17A AF488	BD Biosciences	560221
Anti-CCL2 (MCP-1) FITC	Invitrogen	11-7096-81
Anti-FITC Biotin	Biolegend	408304
Streptavidin FITC	Invitrogen	SA1001

Supplemental Table 1. Antibodies used for flow cytometry
